# Racial and Ethnic Disparities in Receipt of General Anesthesia for Cesarean Delivery

**DOI:** 10.1001/jamanetworkopen.2023.50825

**Published:** 2024-01-09

**Authors:** Caroline Leigh Thomas, Elizabeth M. S. Lange, Jennifer M. Banayan, Yinhua Zhu, Chuanhong Liao, Feyce M. Peralta, William A. Grobman, Barbara M. Scavone, Paloma Toledo

**Affiliations:** 1 Department of Anesthesiology and Critical Care, University of Chicago Medical Center, Chicago, Illinois; 2 Department of Anesthesia, Emory University, Atlanta, Georgia; 3 Department of Anesthesiology, Northwestern University, Chicago, Illinois; 4 Feinberg School of Medicine, Northwestern University, Chicago, Illinois; 5 Department of Public Health Services, University of Chicago, Chicago, Illinois; 6 Department of Obstetrics and Gynecology, The Ohio State University Wexner Medical Center, Columbus; 7 Department of Anesthesia and Critical Care, University of Chicago Medical Center, Chicago, Illinois; 8 Department Obstetrics & Gynecology, University of Chicago Medical Center, Chicago, Illinois; 9 Department of Anesthesiology, Perioperative Medicine and Pain Management, University of Miami Miller School of Medicine, Miami, Florida

## Abstract

**Importance:**

General anesthesia for cesarean delivery is associated with increased maternal morbidity, and Black and Hispanic pregnant patients have higher rates of general anesthesia use compared with their non-Hispanic White counterparts. It is unknown whether risk factors and indications for general anesthesia differ among patients of differing race and ethnicity.

**Objective:**

To evaluate differences in general anesthesia use for cesarean delivery and the indication for the general anesthetic by race and ethnicity.

**Design, Setting, and Participants:**

In this retrospective, cross-sectional, single-center study, electronic medical records for all 35 117 patients who underwent cesarean delivery at Northwestern Medicine’s Prentice Women’s Hospital from January 1, 2007, to March 2, 2018, were queried for maternal demographics, clinical characteristics, obstetric and anesthetic data, the indication for cesarean delivery, and the indication for general anesthesia when used. Data analysis occurred in August 2023.

**Exposure:**

Cesarean delivery.

**Main Outcomes and Measures:**

The rate of general anesthesia for cesarean delivery by race and ethnicity.

**Results:**

Of the 35 117 patients (median age, 33 years [IQR, 30-36 years]) who underwent cesarean delivery, 1147 (3.3%) received general anesthesia; the rates of general anesthesia were 2.5% for Asian patients (61 of 2422), 5.0% for Black patients (194 of 3895), 3.7% for Hispanic patients (197 of 5305), 2.8% for non-Hispanic White patients (542 of 19 479), and 3.8% (153 of 4016) for all other groups (including those who declined to provide race and ethnicity information) (*P* < .001). A total of 19 933 pregnant patients (56.8%) were in labor at the time of their cesarean delivery. Of those, 16 363 (82.1%) had neuraxial labor analgesia in situ. Among those who had an epidural catheter in situ, there were no racial or ethnic differences in the rates of general anesthesia use vs neuraxial analgesia use (Asian patients, 34 of 503 [6.8%] vs 1289 of 15 860 [8.1%]; Black patients, 78 of 503 [15.5%] vs 1925 of 15 860 [12.1%]; Hispanic patients, 80 of 503 [15.9%] vs 2415 of 15 860 [15.2%]; non-Hispanic White patients, 255 of 503 [50.7%] vs 8285 of 15 860 [52.2%]; and patients of other race or ethnicity, 56 of 503 [11.1%] vs 1946 of 15 860 [12.3%]; *P* = .16). Indications for cesarean delivery and for general anesthesia were not different when stratified by race and ethnicity.

**Conclusions and Relevance:**

Racial disparities in rates of general anesthesia continue to exist; however, this study suggests that, for laboring patients who had labor epidural catheters in situ, no disparity by race or ethnicity existed. Future studies should address whether disparities in care that occur prior to neuraxial catheter placement are associated with higher rates of general anesthesia among patients from ethnic and racial minority groups.

## Key Points

**Question:**

Do rates of general anesthesia use for cesarean delivery differ by race or ethnicity?

**Findings:**

In this cross-sectional study including 35 117 patients undergoing cesarean delivery, rates of general anesthesia use were higher among Black and Hispanic individuals. However, among patients who labored with an epidural catheter in situ, there were no significant differences in rates of general anesthesia use by race or ethnicity.

**Meaning:**

This study suggests that use of neuraxial labor analgesia may mitigate racial or ethnic disparities in general anesthesia use for cesarean delivery.

## Teaser

This cross-sectional study evaluates differences in general anesthesia use for cesarean delivery by race and ethnicity.

## Introduction

Widespread adoption of neuraxial analgesia and anesthesia into clinical obstetric anesthesia practice has resulted in a reduction in anesthesia-related maternal morbidity and mortality in the peripartum period.^1,2^ As rates of general anesthesia for cesarean delivery have decreased, there has been a coincident decrease in anesthesia-related morbidity and mortality.^1,3,4^ Compared with neuraxial anesthesia, general anesthesia for cesarean delivery is associated with higher rates of cardiac arrest, aspiration of gastric contents, airway management complications, surgical site infections, postpartum hemorrhage, and maternal mortality.^1,5,6,7,8^ Furthermore, neuraxial anesthesia techniques allow for the administration of neuraxial opioids for postoperative pain control, thus minimizing systemic opioid use and improving maternal ambulation and return of bowel function while also decreasing opioid exposure to the fetus.^5,9,10,11^

Reasons for general anesthesia use in modern clinical practice might include rescue general anesthesia for inadequate neuraxial anesthesia, general anesthesia during emergency circumstances in which there is not time to safely provide neuraxial anesthesia, or patient refusal of neuraxial anesthesia. Reducing rates of potentially avoidable use of general anesthesia has been proposed as an actionable clinical intervention to improve maternal morbidity and outcomes.^12^ Rates of preventable general anesthetics are high (up to 44%) and are associated with anesthetic complications, surgical site infections, and venous thromboembolic events.

Racial disparities exist in the rates of neuraxial labor analgesia use, as well as in the use of neuraxial anesthesia for cesarean delivery.^13,14,15,16^ Non-Hispanic Black (hereafter, Black) and Hispanic patients have higher odds of undergoing general anesthesia for cesarean delivery compared with non-Hispanic White (hereafter, White) patients.^17,18^ There is a paucity of literature addressing why this disparity exists, and most studies evaluating racial and ethnic disparities in anesthetic techniques for cesarean delivery have been performed on a population level, lacking granularity.

Consequently, it is uncertain whether risk factors and indications for general anesthesia for cesarean delivery differ among racial and ethnic groups.^12,18^ Identifying and addressing the cause of racial and ethnic anesthetic disparities may improve maternal outcomes and lessen disparity gaps. The objective of this study was to evaluate differences in general anesthesia use for cesarean delivery and its indications by race and ethnicity.

## Methods

The study was approved by the Northwestern University institutional review board. A waiver of consent was granted for retrospective medical record review. Electronic medical record data for all pregnant patients who underwent a cesarean delivery at Northwestern Medicine’s Prentice Women’s Hospital between January 1, 2007, and March 2, 2018, were evaluated using the Northwestern University Enterprise Data Warehouse. The start date was selected based on the date on which electronic medical record data were first available for neuraxial labor analgesia. The end date was chosen as the date the hospital migrated to a new electronic medical record system. The only exclusion criterion was perimortem cesarean delivery. This report followed the Strengthening the Reporting of Observational Studies in Epidemiology (STROBE) reporting guideline for cross-sectional studies.^19^

Demographic data extracted from the medical record using an electronic medical record query included maternal age, race and ethnicity, body mass index, insurance status (private insurance, Medicaid or public insurance, or unknown insurance status or none), and maternal comorbidities including diagnoses of anemia, diabetes, and hypertensive disorders of pregnancy. Race and ethnicity information was self-identified and provided by the patient on admission. At the time of data entry, there was not an option for a multiracial or multiethnic self-identification.

Race and ethnicity were then queried from the medical record. Data were classified as Asian, Black, Hispanic, White, and other. The “other” category included data for patients with low study representation (ie, American Indian or Alaska Native and Native Hawaiian or Pacific Islander) and patients without information on race or ethnicity.

Obstetric data included parity, gestational age, indication for the cesarean delivery, and whether the patient was laboring prior to the cesarean delivery. Information regarding laboring vs nonlaboring status was obtained from nursing documentation. Information regarding postpartum hemorrhage was queried from delivery notes; postpartum hemorrhage was defined as an estimated blood loss of greater than 1 L. Data regarding maternal comorbidities, such as diabetes or anemia, were obtained from a combination of billing codes from the hospital discharge summary, nursing flowsheets, and physician documentation.

Anesthetic data included the American Society of Anesthesiologists physical status classification, including emergency designation, anesthetic technique for cesarean delivery, and the presence of existing neuraxial labor analgesia. Patients who received both neuraxial and general anesthesia were classified as having received general anesthesia. For patients who received general anesthesia, both the indication for the cesarean delivery and the indication for general anesthesia were manually extracted via medical record review and were categorized using criteria modified from prior studies (eTable in Supplement 1).^18,20^

Manual extraction of the indication for cesarean delivery and the indication for general anesthesia for each patient who underwent general anesthesia was performed by 2 of us (C.L.T. and Y.Z.). Failed neuraxial anesthesia was defined as either a failed initiation of spinal anesthesia or a failed intrapartum extension of an in situ catheter, not in the setting of fetal or obstetric emergency. Neuraxial anesthesia failure could have occurred for a number of reasons (eg, inadequate dermatomal coverage prior to cesarean delivery, intraoperative discomfort necessitating conversion to general anesthesia, or inability to place a spinal or epidural catheter).

Additional data collected included neonatal outcome (live birth vs fetal demise), postpartum hemorrhage, maternal blood transfusion, maternal postoperative intensive care unit admission, and severe maternal morbidity. For severe maternal morbidity, we used a composite of 21 conditions defined by the Centers for Disease Control and Prevention.^21^

The primary outcome was the rate of general anesthesia use stratified by race and ethnicity. Statistical analysis was performed in August 2023. Normal distribution of continuous variables was evaluated with the Shapiro-Wilk test. Categorical data were compared using the Pearson χ^2^ test or the Fisher exact test, and continuous data were compared using the Mann-Whitney test. Univariate and multivariable mixed-effects logistic regression analyses were performed to evaluate the associations between general anesthesia use and all risk factors. Potential risk factors identified in univariate analysis with *P* ≤ .10 were incorporated into a multivariable mixed-effects logistic regression model to create adjusted odds ratios (AORs) for general anesthesia use, in which the random effect was multiple deliveries by the same patient. Variables in the final multivariable mixed-effects logistic regression model included maternal age, race and ethnicity, insurance status, parity, multiple pregnancy, and preeclampsia. All *P* values were from 2-sided tests and results were deemed statistically significant at *P* < .05. Data were analyzed using Stata/SE, version 18 (StataCorp LP).

## Results

A total of 35 117 individuals (median age, 33 years [IQR, 30-36 years]) who underwent cesarean deliveries were identified; none were excluded because of perimortem status. Of patients undergoing cesarean delivery, 2422 were Asian (6.9%), 3895 were Black (11.1%), 5305 were Hispanic (15.1%), 19 479 were White (55.5%), and 4016 (11.4%) had other racial or ethnic identities or did not provide race and ethnicity information (Table 1). Patients who received general anesthesia were more likely than those who received neuraxial anesthesia to have public insurance, be parous, have multiple gestations, and carry a maternal diagnosis of gestational hypertension or preeclampsia. They also had a higher American Society of Anesthesiologists physical status classification and were more likely to be designated as requiring emergency delivery. General anesthesia was performed in 1147 of cases (3.3%). A total of 19 933 pregnant patients (56.8%) were in labor immediately prior to their cesarean delivery. The rates of general anesthesia differed by race and ethnicity and were 2.5% for Asian patients (61 of 2422), 5.0% for Black patients (194 of 3895), 3.7% for Hispanic patients (197 of 5305), 2.8% for White patients (542 of 19 479), and 3.8% for all other patients (153 of 4016) (*P* < .001).

**Table 1.** Patient Characteristics

Characteristic	General anesthesia (n = 1147)^a^	Neuraxial anesthesia (n = 33 970)^a^	*P* value
Demographic data			
Age, median (IQR), y	32 (28-35)	33 (30-36)	<.001
Insurance status, No. (%)			
Private insurance	738 (64.3)	25 261 (74.4)	<.001
Medicaid or public aid	409 (35.7)	8706 (25.6)	
Unknown	0	3 (0.01)	
Race and ethnicity, No. (%)			
Asian	61 (5.3)	2361 (7.0)	<.001
Black	194 (16.9)	3701 (10.9)	
Hispanic	197 (17.2)	5108 (15.0)	
White	542 (47.3)	18 937 (55.7)	
Other^b^	153 (13.3)	3863 (11.4)	
BMI, median (IQR)	29.9 (26.6-34.6)	30.3 (27.3-34.4)	.02
Anesthetic data			
ASA physical status, No./total No. (%)			
2	662/983 (67.3)	24 395/29 132 (83.7)	<.001
3	301/983 (30.6)	4713/29 132 (16.2)	
4	20/983 (2.0)	24/29 132 (0.1)	
5	0	0	
Emergency status, No. (%)	418 (42.5)	6939 (23.8)	<.001
Parity			
Nulliparous, No. (%)	559 (48.7)	18 055 (53.2)	.004
Gestational age, median (IQR), wk	38.5 (34.2-40.0)	39.1 (38.1-40.0)	<.001
No. of gestations			
Multiple gestations, No. (%)	129 (11.3)	2773 (8.2)	<.001
Chorioamnionitis, No. (%)	65 (5.7)	2149 (6.3)	.38
Prior cesarean delivery, No. (%)	144 (12.6)	9472 (27.9)	<.001
Maternal comorbidities, No. (%)			
Anemia	45 (10.1)	929 (8.0)	.13
Diabetes	69 (6.0)	2330 (6.9)	.28
Chronic hypertension	38 (3.3)	888 (2.6)	.16
Gestational hypertension	30 (2.6)	599 (1.8)	.04
Preeclampsia, eclampsia, or HELLP	106 (9.2)	1247 (3.7)	<.001

Abbreviations: ASA, American Society of Anesthesiologists; BMI, body mass index (calculated as weight in kilograms divided by height in meters squared); HELLP, syndrome of hemolysis, elevated liver enzymes, and low platelets.

^a^ Percentages in all categories may not add to 100% due to missing values.

^b^ Includes patients who identify as American Indian or Alaska Native, Native Hawaiian or Pacific Islander, and patients without information on race or ethnicity.

Patients undergoing general anesthesia had higher rates of composite severe maternal morbidity, more intensive care unit admissions, higher rates of fetal demise, and higher rates of blood transfusion (Table 2). After controlling for covariates, Black patients’ odds of undergoing general anesthesia were 1.42 times that of White patients (AOR, 1.42 [95% CI, 1.15-1.75]) (Table 3). Other independent risk factors for general anesthesia included public insurance status (AOR, 1.31 [95% CI, 1.12-1.55]), multiple gestations (AOR, 1.47 [95% CI, 1.19-1.82]), and a diagnosis of preeclampsia (AOR, 2.61 [95% CI, 2.03-3.37]). Older age was a protective factor against general anesthesia (AOR, 0.96 [95% CI, 0.94-0.97]).

**Table 2.** Cesarean Delivery Data

Data	Patients, No. (%)		*P* value
	General anesthesia (n = 1147)^a^	Neuraxial anesthesia (n = 33 970)^a^	
Cesarean description			
No labor	265 (23.1)	13 596 (40.0)	<.001
Labor	851 (74.2)	19 082 (56.2)	
Unknown	31 (2.7)	1288 (3.8)	
Race and ethnicity for patients who were in labor			
Asian	53 (6.2)	1509 (7.9)	<.001
Black	140 (16.5)	2230 (11.7)	
Hispanic	147 (17.3)	2937 (15.4)	
White	398 (46.7)	10 085 (52.9)	
Other^b^	113 (13.3)	2321 (12.2)	
Epidural catheter in situ	503 (59.1)	15 860 (83.1)	<.001
Race and ethnicity for patients who were in labor with an epidural catheter in situ			
Asian	34 (6.8)	1289 (8.1)	.16
Black	78 (15.5)	1925 (12.1)	
Hispanic	80 (15.9)	2415 (15.2)	
White	255 (50.7)	8285 (52.2)	
Other	56 (11.1)	1946 (12.3)	
Race and ethnicity for patients who were in labor without an epidural catheter in situ			
White	143 (41.1)	1796 (55.8)	<.001
Black	62 (17.8)	305 (9.5)	
Hispanic	67 (19.3)	522 (16.2)	
Asian	19 (5.5)	219 (6.8)	
Other	57 (16.4)	374 (11.6)	
Severe maternal morbidity	263 (22.9)	2937 (8.7)	<.001
Asian	14 (5.3)	221 (7.5)	.002
Black	51 (19.4)	427 (14.5)	
Hispanic	55 (20.9)	461 (15.7)	
White	112 (42.6)	1565 (53.3)	
Other	31 (11.8)	263 (9.0)	
ICU admission	115 (10.0)	204 (0.6)	<.001
Asian	6 (5.2)	14 (6.9)	.50
Black	24 (20.9)	55 (27.0)	
Hispanic	22 (19.1)	29 (14.2)	
White	49 (42.6)	88 (43.1)	
Other	14 (12.2)	18 (8.8)	
Birth outcome			
Live birth	1034 (99.1)	31 811 (99.9)	<.001
Fetal demise	9 (0.9)	36 (0.1)	
Postpartum hemorrhage	365 (32.6)	4698 (14.0)	<.001
Asian	18 (4.9)	356 (7.6)	.02
Black	60 (16.4)	570 (12.1)	
Hispanic	65 (17.8)	695 (14.8)	
White	177 (48.5)	2533 (53.9)	
Other	45 (12.3)	543 (11.6)	
pRBC transfusion	83 (7.2)	102 (0.3)	<.001

Abbreviations: ICU, intensive care unit; pRBC, packed red blood cell.

^a^ Percentages in all categories may not add to 100% due to missing values.

^b^ Includes patients who identify as American Indian or Alaska Native, Native Hawaiian or Pacific Islander, and patients without information on race or ethnicity.

**Table 3.** Mixed-Effects Logistic Regression Models

Risk factor	Univariate mixed-effects logistic regression		Multivariable mixed-effects logistic regression	
	OR (95% CI)	*P* value	AOR (95% CI)	*P* value
Age	0.94 (0.93-0.95)	<.001	0.96 (0.94-0.97)	<.001
Insurance status				
Private insurance	1 [Reference]		1 [Reference]	NA
Medicaid or public aid	1.67 (1.46-1.92)	<.001	1.31 (1.12-1.55)	.001
Race and ethnicity				
White	1 [Reference]		1 [Reference]	
Asian	0.90 (0.67-1.20)	.46	0.88 (0.65-1.18)	.39
Black	1.96 (1.61-2.37)	<.001	1.42 (1.15-1.75)	.001
Hispanic	1.40 (1.16-1.68)	<.001	1.02 (0.83-1.25)	.87
Other^a^	1.42 (1.16-1.74)	.001	1.23 (0.99-1.51)	.06
Body mass index	0.99 (0.98-1.01)	.30	NA	NA
Parity				
Parous	1 [Reference]	NA	1 [Reference]	NA
Nulliparous	1.23 (1.08-1.40)	.002	1.12 (0.97-1.28)	.11
Gestational age	0.87 (0.85-0.88)	<.001	NA	NA
No. of gestations				
Singleton	1 [Reference]	NA	1 [Reference]	NA
Multiple gestations	1.48 (1.20-1.82)	<.001	1.47 (1.19-1.82)	<.001
Chorioamnionitis	0.88 (0.67-1.16)	.37	NA	NA
Anemia	1.33 (0.92-1.91)	.13	NA	NA
Diabetes	0.86 (0.66-1.13)	.27	NA	NA
Chronic hypertension	1.30 (0.90-1.88)	.16	NA	NA
Gestational hypertension	1.58 (1.04-2.40)	.03	NA	NA
Preeclampsia				
No preeclampsia	1 [Reference]	NA	1 [Reference]	NA
Preeclampsia, eclampsia, or HELLP	3.00 (2.34-3.86)	<.001	2.61 (2.03-3.37)	<.001
Cesarean description				
No labor	1 [Reference]	NA	NA	NA
Labor	2.49 (2.12-2.92)	<.001	NA	NA
Unknown labor	1.25 (0.83-1.88)	.28	NA	NA
Labor epidural in situ	0.28 (0.23-0.35)	<.001	NA	NA

Abbreviations: AOR, adjusted odds ratio; HELLP, syndrome of hemolysis, elevated liver enzymes, and low platelets; NA, not applicable; OR, odds ratio.

^a^ Includes patients who identify as American Indian or Alaska Native, Native Hawaiian or Pacific Islander, and patients without information on race or ethnicity.

There were racial and ethnic differences between the general anesthesia rates and neuraxial anesthesia rates among patients in labor (eFigure in Supplement 1); however, there were no differences among the laboring patients who had an epidural catheter in situ (Asian patients, 34 of 503 [6.8%] vs 1289 of 15 860 [8.1%]; Black patients, 78 of 503 [15.5%] vs 1925 of 15 860 [12.1%]; Hispanic patients, 80 of 503 [15.9%] vs 2415 of 15 860 [15.2%]; White patients, 255 of 503 [50.7%] vs 8285 of 15 860 [52.2%]; and patients of other race or ethnicity, 56 of 503 [11.1%] vs 1946 of 15 860 [12.3%]; *P* = .16) (Table 2). Of laboring patients, 16 363 (82.1%) had a preexisting epidural catheter in situ at the time of cesarean delivery.

The 3 most common indications for cesarean delivery being performed with general anesthesia were obstetric or fetal emergency, arrest of labor, and maternal hemorrhage (Table 4). The 3 most common indications for general anesthesia use were obstetric or fetal emergency, failed neuraxial anesthesia, and maternal contraindications to neuraxial anesthesia. Neither the indications for cesarean delivery nor the indications for general anesthesia use differed by race or ethnicity.

**Table 4.** Indication for Cesarean Delivery and General Anesthesia by Race and Ethnicity

Indication	Patients, No. (%)					*P* value
	Asian (n = 61)	Black (n = 193)	Hispanic (n = 194)	White (n = 534)	Other (n = 151)^a^	
Indication for cesarean delivery performed with general anesthesia						
Antepartum or intrapartum hemorrhage, abruption, placenta accreta spectrum, previa, or vasa previa	6 (9.8)	22 (11.4)	20 (10.3)	49 (9.2)	20 (13.3)	.13
Arrest of labor, failed induction of labor, or dystocia	13 (21.3)	24 (12.4)	28 (14.4)	102 (19.1)	25 (16.6)	
Breech or malpresentation	7 (11.5)	18 (9.3)	26 (13.4)	34 (6.4)	16 (10.6)	
Deteriorating maternal condition	0	9 (4.7)	7 (3.6)	13 (2.4)	2 (1.3)	
Obstetric or fetal emergency	32 (52.5)	96 (49.7)	98 (50.5)	276 (51.7)	72 (47.7)	
Contraindication to labor	0	7 (3.6)	4 (2.1)	8 (1.5)	0	
High order multiple gestation (or twin A breech)	0	2 (1.0)	2 (1.0)	6 (1.1)	1 (0.7)	
Elective cesarean delivery or planned repeat cesarean delivery	3 (4.9)	15 (7.8)	9 (4.6)	46 (8.6)	15 (9.9)	
Indication for general anesthesia						
Failed neuraxial anesthesia	10 (16.4)	35 (18.1)	37 (19.0)	117 (21.8)	28 (18.4)	.91
Planned intraoperative conversion for additional surgical procedures	3 (4.9)	8 (4.2)	10 (5.1)	24 (4.5)	7 (4.6)	
Unanticipated prolonged operative time	0	3 (1.6)	2 (1.0)	8 (1.5)	3 (2.0)	
Obstetric or fetal emergency	33 (54.1)	104 (53.9)	109 (55.9)	279 (52.1)	75 (49.3)	
Contraindication to neuraxial anesthesia	7 (11.5)	25 (13.0)	19 (9.7)	65 (12.1)	25 (16.5)	
High spinal blockade necessitating respiratory support	1 (1.6)	0	3 (1.5)	7 (1.3)	0	
Deterioration of maternal condition	6 (9.8)	12 (6.2)	13 (6.7)	27 (5.0)	9 (5.9)	
Unknown reason for general despite medical record review	1 (1.6)	3 (1.6)	1 (0.5)	4 (0.8)	4 (2.6)	
Patient preference	0	3 (1.6)	1 (0.5)	5 (0.9)	1 (0.7)	

^a^ Includes patients who identify as American Indian or Alaska Native, Native Hawaiian or Pacific Islander, and patients without information on race or ethnicity.

## Discussion

This study demonstrates that racial and ethnic disparities exist in the use of general anesthesia for cesarean delivery. These findings are consistent with other studies showing that Black patients are nearly twice as likely to undergo general anesthesia for cesarean delivery as White patients.^17,18^ This study extends those findings by demonstrating that there is no racial or ethnic disparity when labor epidural analgesia is provided prior to intrapartum cesarean delivery. The reduction of racial and ethnic disparities among patients with neuraxial catheters in situ for labor is a novel and clinically important finding, as the presence of a catheter in situ for labor allows for the ability to convert labor analgesia to surgical anesthesia for cesarean delivery. In the event that an urgent or intrapartum cesarean delivery is required, timely conversion of neuraxial labor analgesia to anesthesia is one strategy for avoiding a preventable general anesthetic.^22,23^

Disparities do exist in neuraxial labor analgesia use, and Black and Hispanic patients are less likely than White patients to receive neuraxial analgesia.^15,24,25^ Black individuals are less likely to have private insurance and more likely to have no insurance compared with White individuals,^25^ and insurance coverage is associated with significant improvements in access to care, condition-specific outcomes, and self-reported health.^26,27,28^ It is likely that a combination of systemic factors, social determinants of health, clinician factors such as implicit and/or explicit bias, and communication barriers are associated with the disparity in rates of neuraxial analgesia use.^29,30^

While the optimal rate of general anesthesia for cesarean delivery is unknown, national societies such as the Society for Obstetric Anesthesia and Perinatology have established benchmarks and recommendations, with a proposed goal of less than 5% of cesarean deliveries being performed under general anesthesia.^31^ Given that neuraxial labor analgesia may act as a safety mechanism to prevent use of general anesthesia, it is imperative that disparities in neuraxial labor analgesia use be further investigated and addressed. It is possible that early explanation of labor analgesic options and identification of patient preferences in regard to neuraxial analgesia may be key to mitigating disparities in neuraxial labor anesthesia care.^32,33^ In addition, because having an epidural in situ for an intrapartum cesarean delivery was associated with a similar general anesthesia rate stratified by race and ethnicity, future studies may consider evaluating intrapartum vs nonintrapartum cesarean deliveries for obstetrical indications. Nonintrapartum cesarean deliveries might have other actionable factors that should be examined with a health care equity lens.

Given that we manually reviewed each case of an individual receiving general anesthesia, we are able to provide granular data about the clinical indication for the cesarean delivery and general anesthesia. These types of data are challenging to obtain from large databases that rely on administrative data or registries. We found no significant disparities in indications for cesarean delivery performed under general anesthesia or in the indications for general anesthesia.

### Limitations

Our study has some limitations. Due to the single-center nature of the data, the results may not be generalizable to maternal care in the US. The labor analgesia use rate at Northwestern Medicine’s Prentice Women’s Hospital is, on average, above 95%, indicating that the risk of avoidable general anesthesia for our patient cohort may be lower than other institutions where labor analgesia is used less often.^12^ In addition, our data are retrospective; thus, we cannot infer causality. Our data lacked information that could categorize the urgency of cesarean delivery, which precluded our ability to evaluate general anesthetics for preventability. Because this study investigates only patients who underwent cesarean delivery, we are unable to provide rates of labor analgesia by race and ethnicity for all laboring patients at our institution.

## Conclusions

These findings have both clinical and public health implications. The data suggest that the racial and ethnic disparities in general anesthesia rates exist in association with neuraxial catheter placement and that once a neuraxial catheter is in situ, these disparities no longer existed for the subset of patients undergoing intrapartum cesarean delivery. We speculate that the cause of this finding may be complex and may involve both patient-related and clinician-related factors. Future studies are needed to further elucidate the cause of the discrepancy in the administration of general anesthesia and neuraxial analgesia and strategies to eliminate it. Attention should focus on patient-centered, timely administration of neuraxial labor analgesia and on identifying actionable items among patients without epidural labor analgesia. In addition, future studies should attempt to replicate our findings to improve generalizability. These findings underscore the need to identify modifiable risk factors for general anesthesia use, to mitigate risk.

## Notes

Supplement 1. **eTable.** Indications for Cesarean Delivery and General Anesthesia **eFigure.** Rates of General Anesthesia in Laboring Patients by Race and Ethnicity

Supplement 2.
Data Sharing Statement
