# Supplementary material for: Racial and Ethnic Disparities in Receipt of General Anesthesia for Cesarean Delivery
Source: JAMA Netw Open. 2024 Jan 9;7(1):e2350825. doi: 10.1001/jamanetworkopen.2023.50825 (PMC10777252; doi:10.1001/jamanetworkopen.2023.50825)
Supplement: Supplement 1. — eTable. Indications for Cesarean Delivery and General Anesthesia eFigure. Rates of General Anesthesia in Laboring Patients by Race and Ethnicity [file jamanetwopen-e2350825-s001.pdf]

Supplementary Online Content

Thomas CL, Lange EMS, Banayan JM, et al. Racial and ethnic disparities in receipt of general anesthesia for cesarean delivery. *JAMA Netw Open.* 2024;7(1):e2350825. doi:10.1001/jamanetworkopen.2023.50825

eFigure. Rates of General Anesthesia in Laboring Patients by Race and Ethnicity

This supplementary material has been provided by the authors to give readers additional information about their work.

© 2024 Thomas CL et al. *JAMA Network Open.*

eFigure. Rates of General Anesthesia in Laboring Patients by Race and Ethnicity

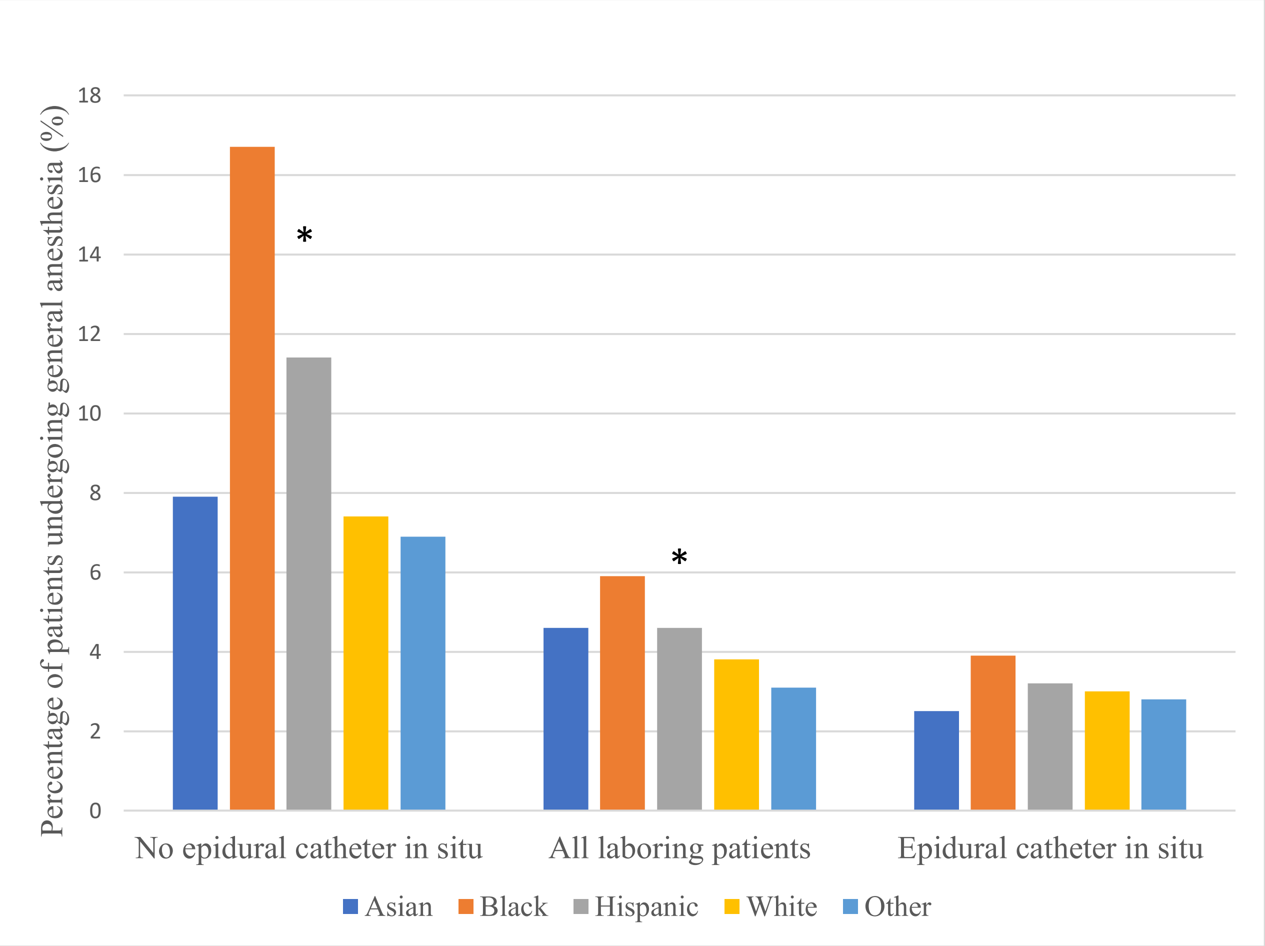

Rates of general anesthesia in laboring patients by race and ethnicity. * *P*<0.001. Other category includes subjects who identify as Native American or Alaskan Native, Native Hawaiian or Pacific Islander), and subjects without information on race or ethnicity.

© 2024 Thomas CL et al. *JAMA Network Open*.
