# Supplementary material for: Racial and Ethnic Disparities in Receipt of General Anesthesia for Cesarean Delivery
Source: JAMA Netw Open. 2024 Jan 9;7(1):e2350825. doi: 10.1001/jamanetworkopen.2023.50825 (PMC10777252; doi:10.1001/jamanetworkopen.2023.50825)
Supplement: Supplement 2. — Data Sharing Statement [file jamanetwopen-e2350825-s002.pdf]

Data Sharing Statement

Thomas. Racial and Ethnic Disparities in Receipt of General Anesthesia for Cesarean Delivery. *JAMA Netw Open*. Published January 08, 2024. doi:10.1001/jamanetworkopen.2023.50825

Data
Data available: No
